# Supplementary material for: DNA replication initiation in Bacillus subtilis: structural and functional characterization of the essential DnaA–DnaD interaction
Source: Nucleic Acids Res. 2018 Dec 8;47(4):2101–12. doi: 10.1093/nar/gky1220 (PMC6393240; doi:10.1093/nar/gky1220)
Supplement: Supplementary Data [file gky1220_supplemental_files.pdf]

SUPPLEMENTARY MATERIAL

DNA replication initiation in *Bacillus subtilis*; Structural and functional characterisation of the essential DnaA-DnaD interaction

Eleyna Martin[1], Huw E. L. Williams[1], Matthaios Pitoulias[1], Daniel Stevens[2], Charles Winterhalter[2], Timothy D. Craggs[3], Heath Murray [2], Mark S. Searle[1,*], Panos Soultanas [1,*]

[1]Centre for Biomolecular Sciences
School of Chemistry
University of Nottingham
Nottingham NG7 2RD
UK

[2]Centre for Bacterial Cell Biology
Medical School
Newcastle University
Newcastle NE2 4AX
UK

[3]School of Chemistry
University of Sheffield
Western Bank
Sheffield S10 2TN
UK

*Joint corresponding authors
Panos.Soultanas@nottingham.ac.uk
Mark.Searle@nottingham.ac.uk

B. subtilis 168 DnaA: 446 aa.
MENILDLWNQALAQIEKKLSKPSFETWMKSTKAHSLQGDTLTITAPNEFARDWLESRYLHLIADTIY
ELTGEELSIKFVIPQNQDVEDFMPKPQVKKAVKEDTSDFPQNMLNPKYTFDTFVIGSGNRFAHAAS
LAVAEAPAKAYNPLFIYGGVGLGKTHLMHAIGHYVIDHNPSAKVVYLSSEKFTNEFI**N**SIRDNK**A**V
DFRNRYRNVDVLLIDDIQFLAGKEQTQEEFFHTFNTLHEESKQIVISSDRPPKEIPTLEDRLRSRFE
WGLITDITPPDLETRIAILRKKAKAEGLDIPNEVMLYIANQIDSNIRELEGALIRVVAYSSLINKDINADL
AAEALKDIIPSSKPKVITIKEIQRVVGQQFNIKLEDFKAKKRTKSVAFPRQIAMYLSREMTDSSLPKIG
EEFGGRDHTTVIHAHEKISKLLADDEQLQQHVKEIKEQLK

B. subtilis 168 DnaD: 232 aa.

MKKQQFIDMQEQGTSTIPNLLLTHYKQLGLNETELILLLKIKMHLEKGSYFPTPNQLQEGMSISVEE
CTNRLRMFIQKGFLFIEECEDQNGIKFEKYSLQPLWGKLYEYIQLAQNQTQERKAEGEQKSLYTIFE
EEFARPLSPLECETLAIWQDQDQHDAQLIKHALKEAVLSGKLSFRYIDRILFEWKKNGLKT**V**EQAKI
HSQKFRRVQAKQNEPQKEYKRQVPFYNWLEQ

Suppl. Fig. S1

The amino acid sequences of *B. subtilis* DnaA and DnaD. The DnaA domain I (M1-P81, yellow) used in this study is coloured in yellow while the AAA+ domain (F106-A338, green) and C-terminal domain (A339-K446, red) that binds dsDNA are coloured in green and red, respectively. N191 and A198 that were mutated to cysteines to produce the DnaA(N191C/A198C) protein are shown in magnified bold. The N-terminal (M1-S128) and C-terminal (K129-Q232) domains of DnaD containing DDBH1 and DDBH2, respectively, are shown in grey and cyan. V196 which defines the C-terminal end of the truncated DnaD196 protein is shown in magnified bold.

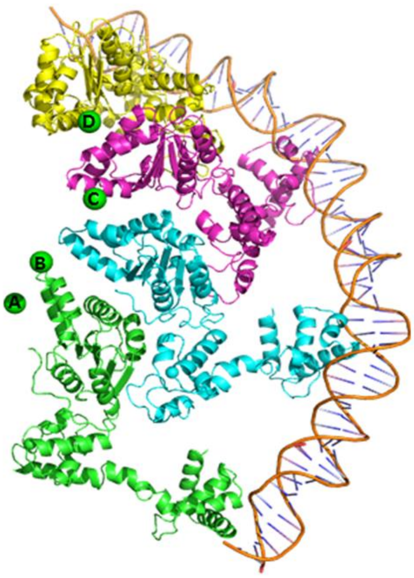

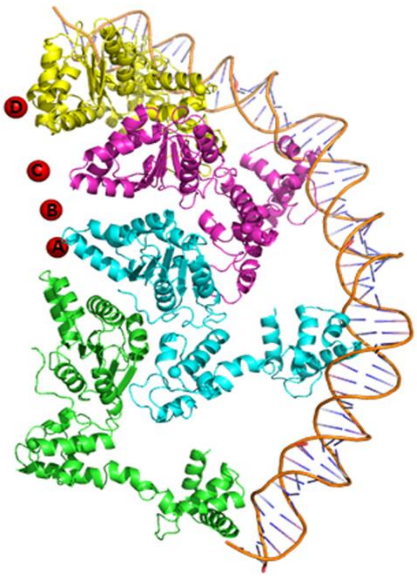

N191	A	B	C	D
A		28.2	50.0	64.3
B			24.2	41.1
C				20.6

A198	A	B	C	D
A		15.0	29.6	48.8
B			14.6	34.1
C				19.7

Suppl. Fig. S2

A mini DnaA(N191C/A198C)-DNA filament model based upon a model reported before (**1**) to assess the interatomic distances of residues N191C and A198C within the filament and the feasibility of FRET experiments using these residues. Relevant interatomic distances in nm are shown in the tables underneath the mini-filaments. Four DnaA molecules are coloured green, cyan, purple and yellow. Each DnaA molecule lacks domains I and II and only domains III and IV are shown.

DNA B. subtilis origin (819 bp)

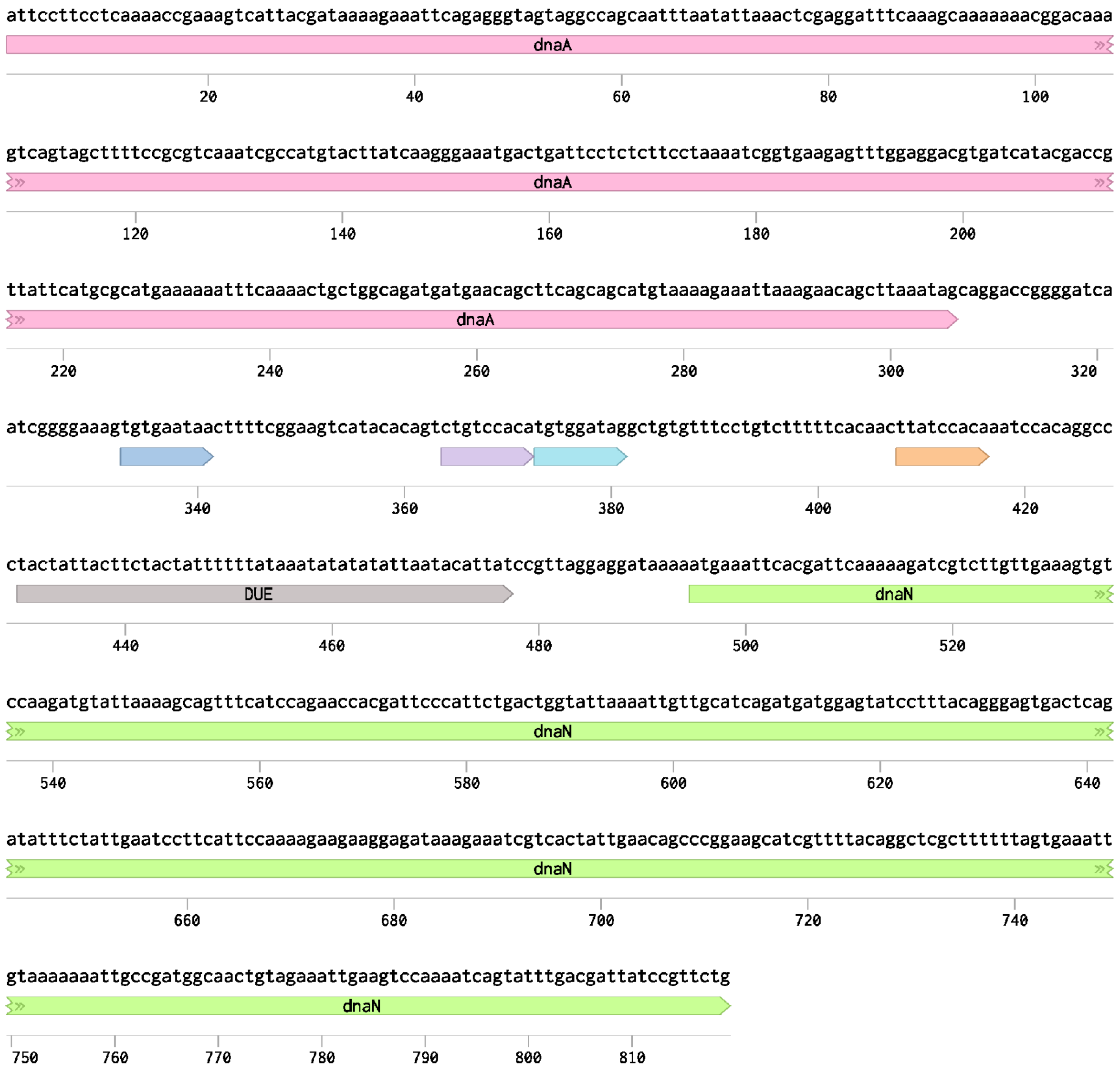

Suppl. Fig. S3

The sequence of the 819 bp DNA fragment, between the *dnaA* and *dnaN* genes containing the half origin with the DUE and four DNA boxes (blue, violet, cyan and orange), used in this study to form DnaA-DNA filaments.

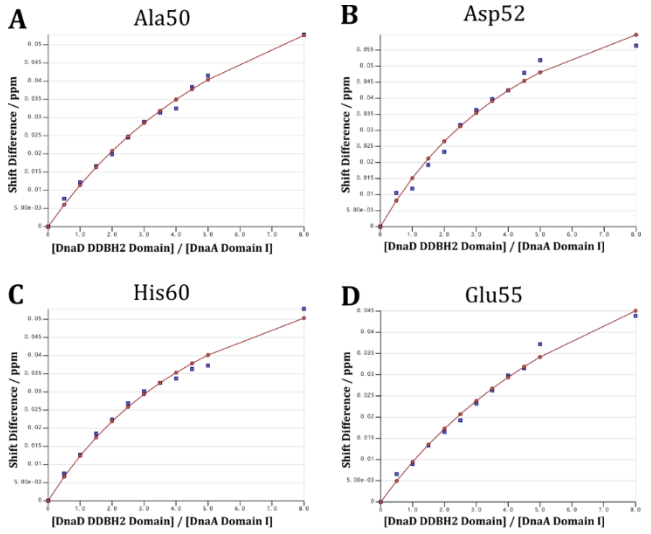

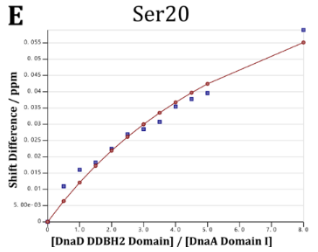

Peak	K_D/ µM	Error K_D/ µM
Ala50	701	85
Asp52	454	99
His60	491	78
Glu55	789	138
Ser20	686	197

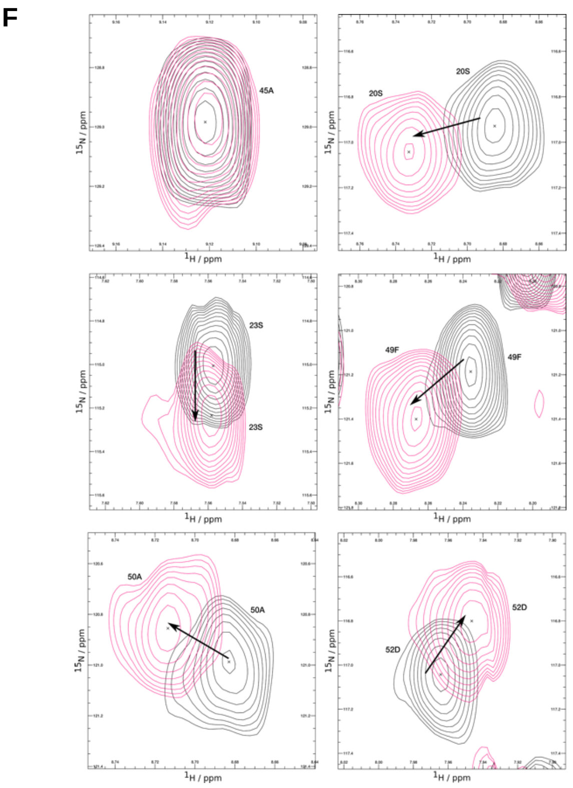

Suppl. Fig. S4

The non-linear fit for the chemical shift changes of residues **A.** Ala50, **B.** Asp52, **C.** His60, **D.** Glu55 and **E.** Ser20 for the DnaD DDBH2 domain titration against ^{15}N-labelled DnaA domain I (100 µM). Experimental data are shown in blue and the fit shown in red. The following equation was used to fit shift differences observed at each ligand concentration ‘$y = A((B + x) - \sqrt{(B + x)^2 - 4x})$’ available as a fitting function within the CCNP Analysis software (2). Where A = (maximum chemical shift difference)/2, $B = 1 + K_D$ / [DnaA domain I], x = [DnaD DDBH2 domain] / [DnaA domain I] and y = chemical shift difference. The residues shown were selected from those that experienced the largest CSP during the titration experiments and provided the best fit to the equation (determined by the error for the calculated K_D value). The mean plus standard deviation across the calculated K_D has a value of 768 µM with an error of 168 µM. **F.** Examples of overlays of spectral peaks representing DnaA domain I (100 µM) ^{15}N HSQC in black and the 8:1 excess of DnaD DDBH2 domain (800 µM) to DnaA domain I spectrum is shown in pink. The arrows indicate direction of movement of the peak during the titration series. The alanine 45 peak (top left) has been included to illustrate the behaviour of peaks not involved in the binding interface.

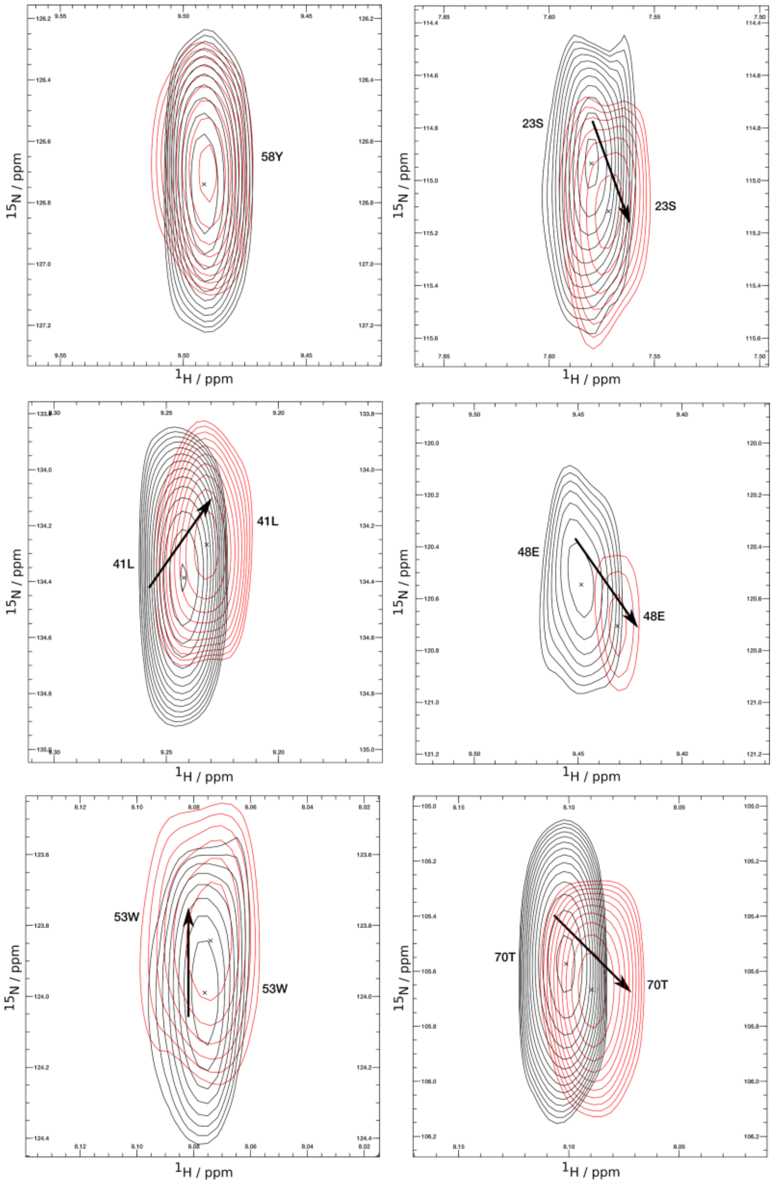

Suppl. Fig. S5

Examples of overlay of spectral peaks representing DnaA domain I (100 µM) ^{15}N HSQC in black and the 8:1 excess of DnaD DDBH2 domain (800 µM) to DnaA domain I spectrum is shown in red, both spectra were run in the presence of ssDNA (800µM). The arrows indicate direction of movement of the peak during the titration series. The tyrosine 58 peak (top left) has been included to illustrate the behaviour of peaks not involved in the binding interface.

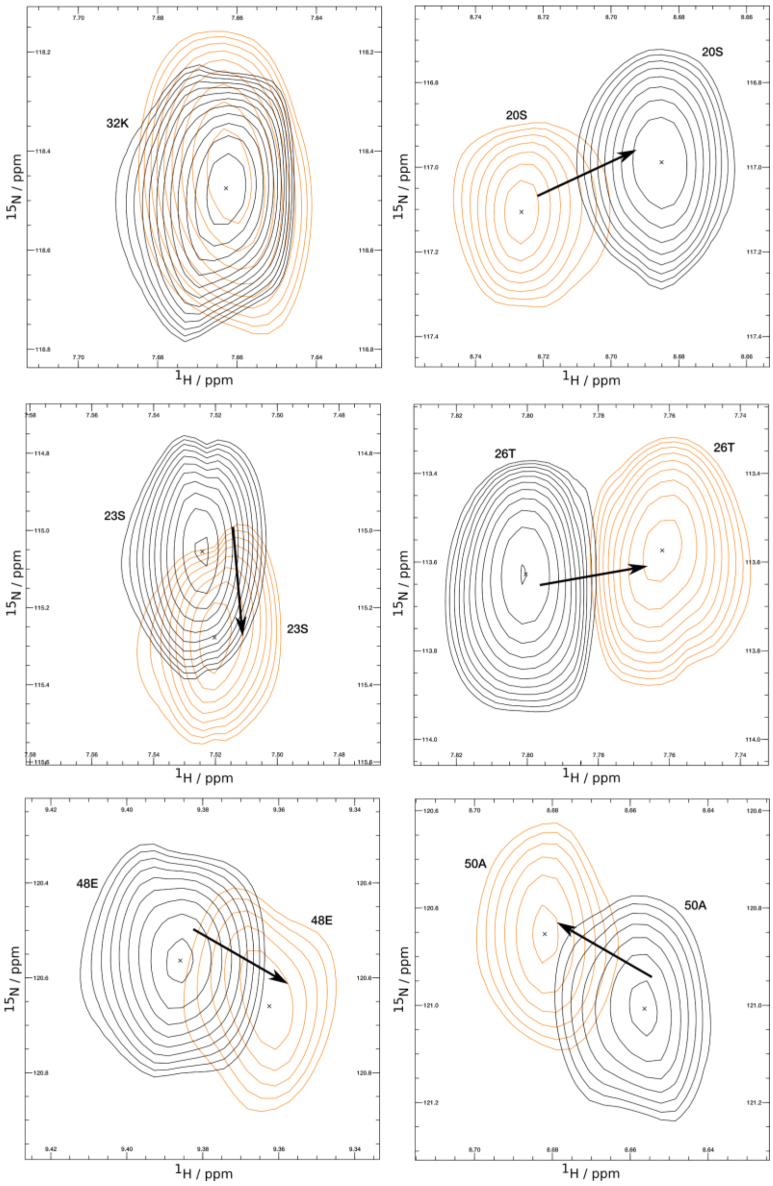

Suppl. Fig. S6

Examples of overlay of spectral peaks representing DnaA domain I (100 µM) ^{15}N HSQC in black and the 8:1 excess of DnaD DDBH2 domain truncation (residues 129 – 196, 800 µM) to DnaA domain I spectrum is shown in orange. The arrows indicate direction of movement of the peak during the titration series. The lysine 32 peak (top left) has been included to illustrate the behaviour of peaks not involved in the binding interface.

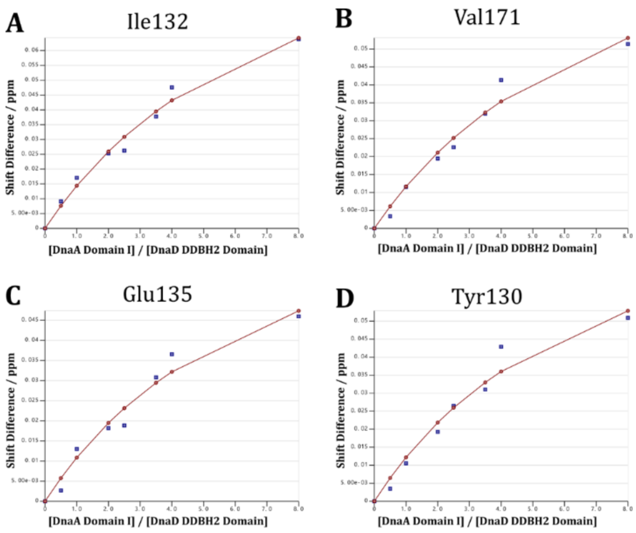

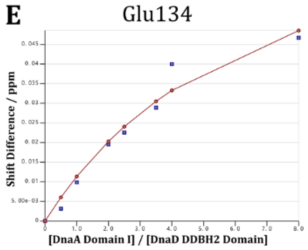

Peak	K_D/ µM	Error K_D/ µM
Ile132	648	188
Val171	684	248
Glu135	603	239
Tyr130	590	237
Glu134	568	230

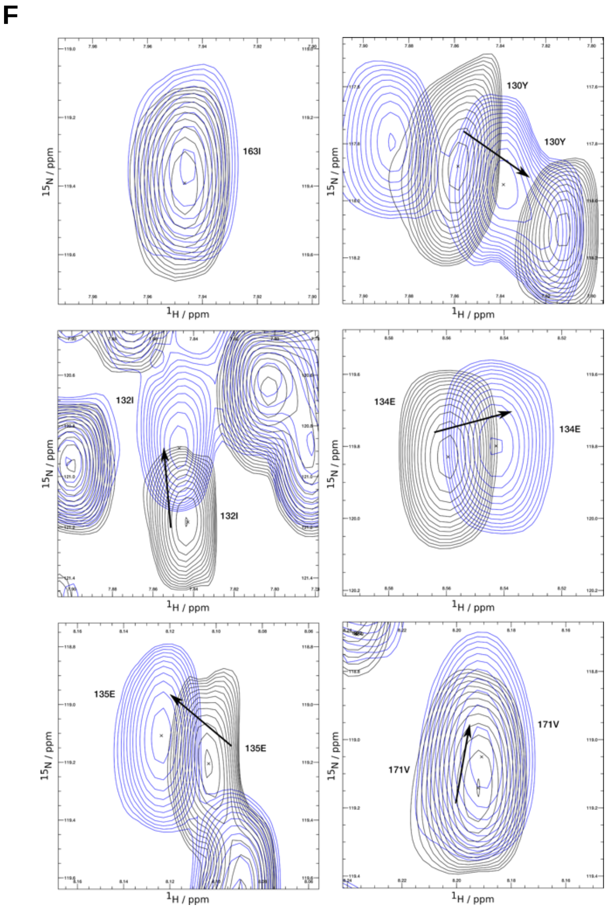

Suppl. Fig. S7

The non-linear fit for the chemical shift changes of residues **A.** Ile132. **B.** Val171, **C.** Glu135, **D.** Tyr130 and **E.** Glu134 for the DnaA domain I titration against the DnaD DDBH2 domain (100 µM). Experimental data are shown in blue and the fit shown in red. The following equation was used to fit shift differences observed at each ligand concentration '$y = A((B + x) - \sqrt{(B + x)^2 - 4x})$' available as a fitting function within the CCNP Analysis software (2). Where A = (maximum chemical shift difference)/2, $B = 1 + K_D$ / [DnaD DDBH2], x = [DnaA domain I] / [DnaD DDBH2 domain] and y = chemical shift difference. The residues shown were selected from those that experienced the largest CSP during the titration experiments and provided the best fit to the equation (determined by the error for the calculated K_D value). The mean plus standard deviation across the calculated K_D has a value of 665 µM with an error of 251 µM. **F.** Examples of overlay of spectral peaks representing DnaD DDBH2 domain (100 µM) ^{15}N HSQC in black and the 8:1 excess of DnaA domain I (800 µM) to DnaA domain I spectrum is shown in blue. The arrows indicate direction of movement of the peak during the titration series. The isoleucine 163 peak (top left) has been included to illustrate the behaviour of peaks not involved in the binding interface.

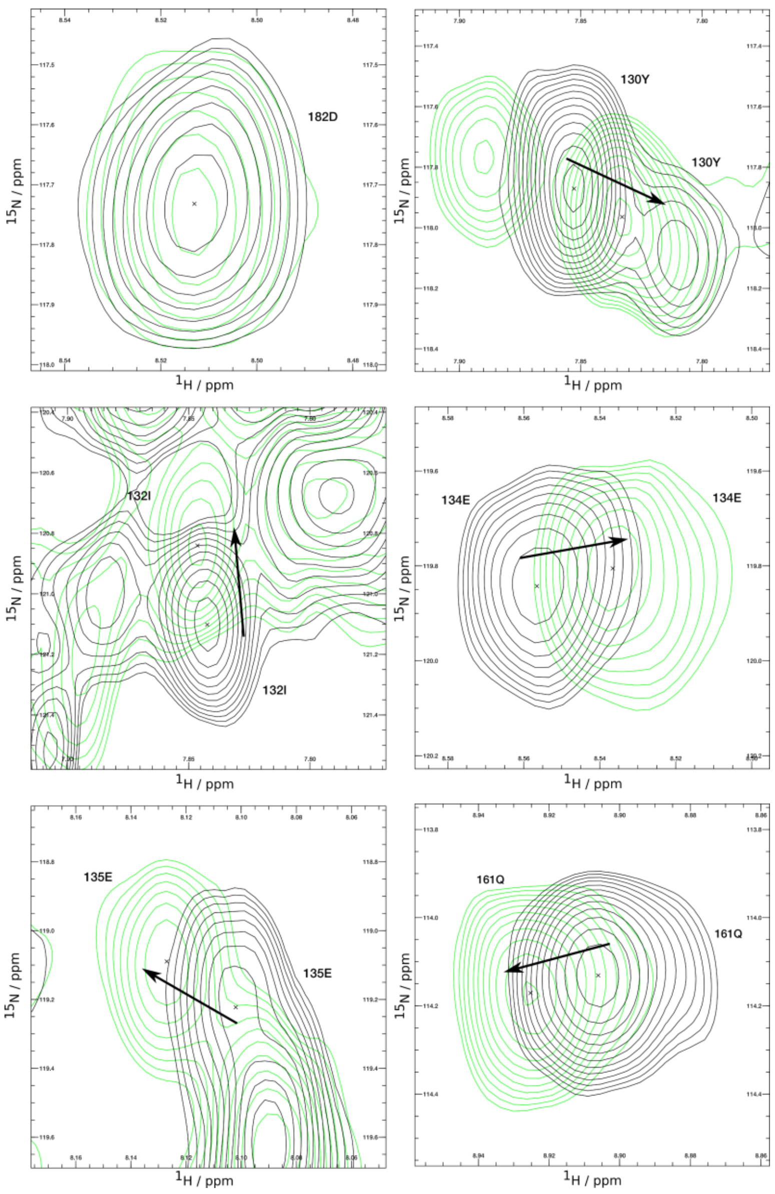

Suppl. Fig. S8

Examples of overlay of spectral peaks representing DnaD DDBH2 domain (100 µM) ^{15}N HSQC in black and the 8:1 excess of DnaA domain I (800 µM) to DnaA domain I spectrum is shown in green, both spectra were run in the presence of ssDNA (800µM). The arrows indicate direction of movement of the peak during the titration series. The aspartate 182 peak (top left) has been included to illustrate the behaviour of peaks not involved in the binding interface.

Parameter	Cluster 1	Cluster 2
HADDOCK Score	-103.5 ±7.0	-107.1 ±23.8
Cluster Size	14	6
RMSD	6.2 ±0.4	1.9 ±1.3
Van der Waals	-33.3 ±4.8	-50.1 ±11.1
Electrostatics	-267.5 ±11.1	-243.2 ±34.8
Desolvation	-34.2 ±3.0	-22.1 ±8.5
Restraint Violation	175.8 ±27.80	136.6 ±36.24
Buried Surface Area	1404.8 ±67.9	1642.4 ±147.5
Z-score	-1.9	-1.9

Suppl. Fig. S9

Scoring parameters for the top 2 clusters generated by HADDOCK restraint-driving docking of the DnaA domain I – DnaD DDBH2 domain interface. The RMSD is calculated from the overall lowest-energy structure within the cluster. The Z-score indicates how many standard deviations from the average the cluster is located in terms of score (the more negative the better).

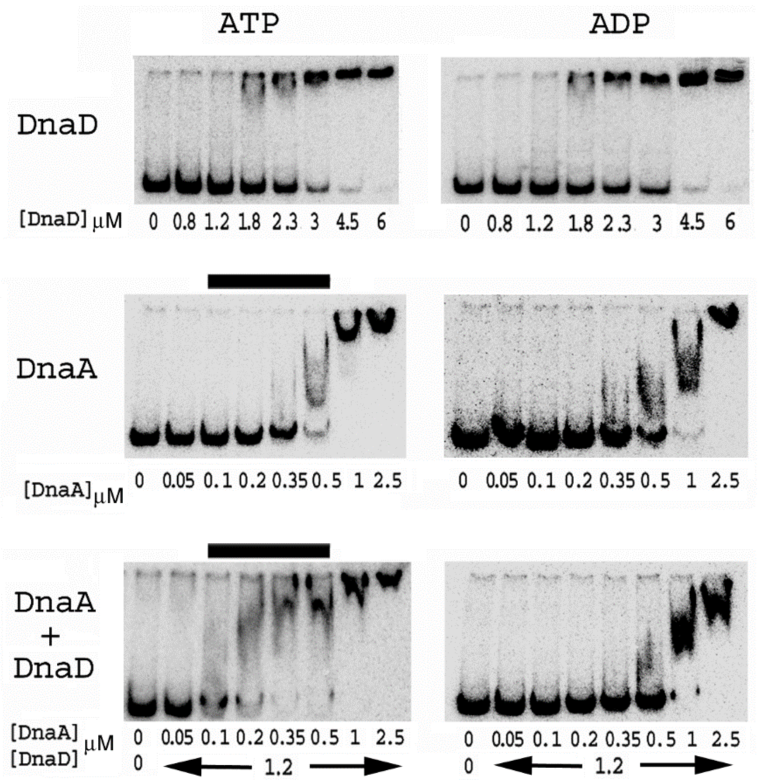

Suppl. Fig. S10

EMSA with increasing concentrations of DnaA (0-2.5 µM) in the presence of a 120 mer ds synthetic oligonucleotide carrying four DnaA boxes (see **Suppl. Fig. S3**), ATP (1 mM) or ADP (1 mM) and in the presence or absence of DnaD (1.2 µM), as indicated. Control EMSA with increasing concentrations of DnaD (0-6 µM) in the presence and absence of ATP (1 mM) or ADP (1 mM) (top two gels) show that at 1.2 µM DnaD does not form a detectable nucleoprotein complex. DnaA forms detectable nucleoprotein complexes in the presence of ATP or ADP but when DnaD is added in preformed DnaA-DNA complexes they appear to change in the presence of ATP but not in the presence of ADP. In both cases there is no detectable disassembly of DnaA-DNA filaments when DnaD is added.

Construct	Primer	Sequence 5′–3′	Ta (°C)
DnaD 129-196	Forward	CGCGGCAGC**catatg**CTTTATACCATTTTTGAG	72
	Reverse	TGCCTGCTC**aagcttta**CACAGTTTTAAGCCC	
DnaA 1-81	Forward	GATATACC**catatg**GAAAATATATTAGACCTG	63
	Reverse	GGCCGC**aagcttttta**AGGAATGACAAACTTAATGC	
DnaD 1-196-his	Forward	GCGGCCGCACTCGAGCAC	71
	Reverse	CACAGTTTTAAGCTTATTTTTCTTCCATTCAAACAAAATCC	

Supp. Fig. S11

DNA oligonucleotide sequences used for PCR and cloning of *B. subtilis* DnaA and DnaD constructs. Insertion mutations are indicated in lowercase bold. Ta values shown correspond to the specific primer annealing temperature used during the PCR reactions.

Construct	Primer	Sequence 5'–3'	Ta (°C)
S75A	Forward	GGAAGAATTG**gcc**ATTAAGTTTGTCATTCC	58
	Reverse	CCGGTTAATTCATATATAGTATC	
T26A	Forward	GAGTTTTGAG**gcc**TGGATGAAGTC	58
	Reverse	GGTTTGCTCAACTTTTTTTC	
S56A	Forward	CTGGCTGGAG**gcc**AGATACTTGC	68
	Reverse	TCTCTGGCAAATTCATTGGGAGC	
T70A	Forward	ATATGAATTA**gcc**GGGGGAAGAATTG	61
	Reverse	CTGGCTGGAGGCCAGATACTTGC	
S23A	Forward	GAGCAAACCG**gcc**TTGAGACTTGG	57
	Reverse	AACTTTTTTTCGATTTGAGC	
E73N	Forward	AACCGGGGAA**aac**TTGAGCATTAAG	60
	Reverse	AATTCATATATAGTATCTGCAATCAG	
R57K	Forward	GCTGGAGTCC**aaa**TACTTGCATC	62
	Reverse	CAGTCTCTGGCAAATTCATTG	
E72N	Forward	ATTAACCGGG**aac**GAATTGAGCATTAAG	59
	Reverse	TCATATATAGTATCTGCAATCAG	
G71A	Forward	TGAATTAACC**gcc**GAAGAATTGAGCATTAAG	59
	Reverse	TATATAGTATCTGCAATCAGATG	
L54A	Forward	CAGAGACTGG**gcc**GAGTCCAGATACTTG	60
	Reverse	GCAAATTCATTGGGAGCC	
E68N	Forward	TACTATATAT**aac**TTAACCGGGGAAGAATTG	59
	Reverse	TCTGCAATCAGATGCAAG	
L74N	Forward	GGGGAAGAA**gct**AGCATTAAGTTTG	56
	Reverse	GTTAATTCATATATAGTAATCTGC	
G38A	Forward	CTCACTGCAA**gcc**GATACATTAAC	61
	Reverse	TGGGCTTTGGTTGACTTC	
ΔM1-L5	Forward	GACCTGTGGAACCAAGCC	67
	Reverse	CATATATGGCTGCCGCGC	
W53A	Forward	TGCCAGAGAC**gcc**CTGGAGTCCAG	64
	Reverse	AATTCATTGGGAGCCGTG	
F49A	Forward	TCCCAATGAA**gcc**GCCAGAGACTG	59
	Reverse	GCCGTGATTGTTAATGTATC	
D52A	Forward	ATTTGCCAGA**gcc**TGGCTGGAGT	66
	Reverse	TCATTGGGAGCCGTGATTG	

Suppl. Fig. S12

Oligonucleotides used for mutagenesis of the DnaA domain I during NMR structure determination. Substitution mutations are indicated in lower bold. Ta values shown correspond to the specific primer annealing temperature used during the PCR reactions.

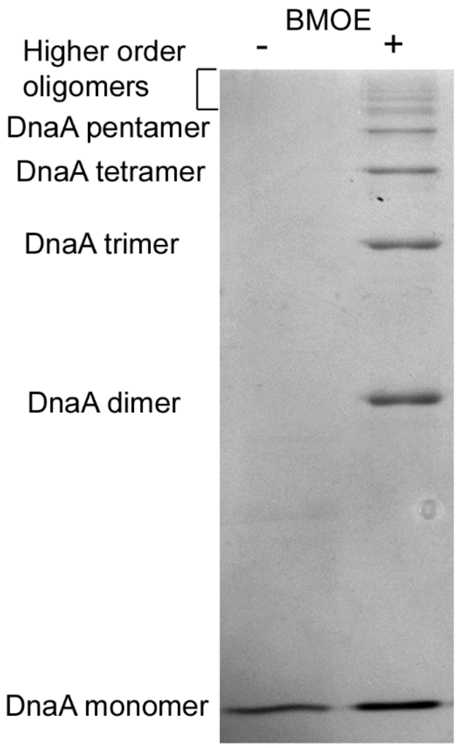

Suppl. Fig. S13

Formation of DnaA(N191C/A198C)-DNA filaments *in vitro*. A 7.5% w/v SDS-PAGE gel showing the DnaA monomer in the absence of BMOE and also in the presence of BMOE. Detailed experimental conditions are described in the Materials and Methods section of the main paper.

AIR Active Residues	
**DnaA Domain I**	**DnaD DDBH2 Domain**
K17	L129
S20	Y130
S23	I132
T26	E134
E48	E135
F49	K164
D52	V171
W53	
E55	
S56	
H60	

Suppl. Table S1

Ambiguous interaction restraints used with the HADDOCK program to model the DnaA domain I – DnaD DDBH2 domain interaction. NMR chemical shift perturbation data was analysed to identify significant residues and solvent exposed residues from this set were designated active residues. Passive residues were determined automatically by the HADDOCK program (solvent exposed neighbours of active residues).

A

Position	Residue	¹H/ppm	¹⁵N/ppm	Position	Residue	¹H/ppm	¹⁵N/ppm	Position	Residue	¹H/ppm	¹⁵N/ppm
2	Glu	8.27635	119.762	29	Lys	7.38332	120.128	56	Ser	7.66342	110.193
3	Asn	8.31644	118.495	30	Ser	7.94273	109.937	57	Arg	7.87877	117.439
4	Ile	8.82464	117.806	31	Thr	7.60675	112.855	58	Tyr	9.48555	126.744
5	Leu	7.77913	119.996	32	Lys	7.71593	118.533	59	Leu	7.33177	123.559
6	Asp	7.74535	119.623	33	Ala	8.41544	124.902	60	His	8.1151	115.752
7	Leu	7.78706	123.392	34	His	8.98387	124.554	61	Leu	7.66262	121.272
8	Trp	8.65123	122.927	35	Ser	7.66444	107.927	62	Ile	8.53303	121.792
9	Asn	7.04753	115.632	36	Leu	8.73621	124.188	63	Ala	9.01167	123.449
10	Gln	8.24587	120.639	37	Gln	8.29228	126.042	64	Asp	8.94856	119.557
11	Ala	8.53478	124.228	38	Gly	7.75757	116.03	65	Thr	7.93182	118.004
12	Leu	8.6606	118.532	39	Asp	8.37905	125.125	66	Ile	8.61053	121.156
13	Ala	7.38361	119.542	40	Thr	7.98033	114.865	67	Tyr	8.61793	125.859
14	Gln	7.38702	115.594	41	Leu	9.23992	134.357	68	Glu	8.33698	121.371
15	Ile	8.75647	122.863	42	Thr	8.95423	123.168	69	Leu	8.54945	118.467
16	Glu	8.33372	121.093	43	Ile	8.98882	129.865	70	Thr	8.08363	105.134
17	Lys	6.75139	112.517	44	Thr	9.31033	121.854	71	Gly	8.76972	112.377
18	Lys	7.68636	117.233	45	Ala	9.11705	128.748	72	Glu	7.28995	112.473
19	Leu	7.23269	119.421	47	Asn	7.1493	107.655	73	Glu	8.72761	123.008
20	Ser	8.74964	116.977	48	Glu	9.43664	120.519	74	Leu	8.13344	126.063
21	Lys	9.14403	123.393	49	Phe	8.27117	121.416	75	Ser	8.90488	117.873
23	Ser	7.57202	114.757	50	Ala	8.72132	120.806	76	Ile	8.25686	125.141
24	Phe	9.95849	128.908	51	Arg	8.1906	118.096	77	Lys	8.29942	126.897
25	Glu	8.48574	119.142	52	Asp	8.04719	117.191	78	Phe	8.88864	120.181
26	Thr	7.82494	113.763	53	Trp	7.98616	123.994	79	Val	8.84919	117.507
27	Trp	7.50955	116.73	54	Leu	7.80942	115.201	80	Ile	7.6846	116.294
28	Met	8.21391	118.933	55	Glu	7.75656	115.752				

^{1}H and ^{15}N chemical shifts for the DnaA domain I amide backbone assignment.

B

Position	Residue	Cα [i] ^{13}C/ppm	Cβ [i] ^{13}C/ppm	CO [i] ^{13}C/ppm	Cα [i-1] ^{13}C/ppm	Cβ [i-1] ^{13}C/ppm	CO [i-1] ^{13}C/ppm	Position	Residue	Cα [i] ^{13}C/ppm	Cβ [i] ^{13}C/ppm	CO [i] ^{13}C/ppm	Cα [i-1] ^{13}C/ppm	Cβ [i-1] ^{13}C/ppm	CO [i-1] ^{13}C/ppm
2	Glu							42	Thr	59.00623	65.80369	170.67176	50.83218	41.36162	170.8429
3	Asn							43	Ile	55.49474	36.96275	171.77595	58.95771	65.72578	170.66484
4	Ile							44	Thr	59.06465	66.77748	171.26885	55.44952	37.11935	171.82246
5	Leu				54.21364			45	Ala	45.47912	17.50562	173.25379	59.12585	66.54761	171.31889
6	Asp	54.35454	33.99764		55.39943	38.41024	176.76876	47	Asn	49.51949	36.97485	171.83478	62.08948	29.64204	174.01122
7	Leu	55.54323			61.38221			48	Glu	57.63284			49.57491	37.03695	171.86953
8	Trp	59.93362	25.86369	172.8519			173.20826	49	Phe	58.10375		175.84999	57.58611	27.04451	174.02855
9	Asn							50	Ala	52.10388	15.77309	175.74355	58.27108	36.07868	175.98239
10	Gln	56.4256	25.8035	176.1379	53.68801	34.97193	176.8193	51	Arg	57.77366	26.87214	174.09341	52.11838	15.89409	175.75894
11	Ala	52.51945	14.9322	176.92977	56.43044	26.06653	176.19367	52	Asp	54.25107	37.52621		57.80689		174.15647
12	Leu	55.27966	39.07714	175.83755	52.53655	15.15471	176.97487	53	Trp	57.98889		175.07567	54.27039	37.75519	176.10304
13	Ala	52.04585	15.00389	177.7615	55.38007	39.1665	175.93624	54	Leu	54.20577		175.47326	54.08799	28.19884	175.54527
14	Gln	55.04335	25.74194	176.69006	51.97825	15.20396	177.8015	55	Glu		41.50972		56.79995	26.67899	
15	Ile	63.16017	35.65962	174.89375	55.05713	25.99929	176.75666	56	Ser				56.90654	27.56773	
16	Glu	56.65141	26.27966	174.73308	63.09105	35.46787	174.9413	57	Arg	54.04949			57.51647	60.99891	
17	Lys	54.56408	29.73506	174.90589	56.42575	26.35325	174.74338	58	Tyr						181.82883
18	Lys	53.96341	31.94171	173.08893	54.47261	29.97348	174.97407	59	Leu	57.70291	39.96496	175.25125	37.52184		172.84113
19	Leu	50.71153	43.56287	174.19566	53.912	32.16839	173.14342	60	His	57.18583	26.21492	174.1899	57.69181	40.09149	175.30116
20	Ser	55.35918	61.37984	171.87315	50.73731	43.51401	174.25869	61	Leu	55.3197	39.11404	177.99827	56.87356	26.35765	174.27929
21	Lys	55.35313			55.32898	61.51587	171.9216	62	Ile	63.81559	35.65229	174.45666	55.3202	39.24646	178.04781
23	Ser	59.54429			62.96017		177.52259	63	Ala	53.79127	15.33392	178.37259	63.86103	35.59181	174.51527
24	Phe						181.01941	64	Asp	54.75463	37.81812	176.08486	53.80679	15.55222	178.40836
25	Glu	56.01297	27.59768	174.38649	55.67482	38.97921	175.32101	65	Thr	64.6396	66.01002	173.48927	54.87012	37.89152	176.13825
26	Thr	63.36459	66.24896	172.55631	56.65004	27.1381	175.15108	66	Ile	59.8806	32.85607	176.78828	64.73846	65.95142	173.54425
27	Trp	54.71074		173.75689	63.33057	65.98634	172.59861	67	Tyr	58.38405	35.25648	176.0372	59.87887	32.79555	176.85274
28	Met	51.73264			43.0086		170.2734	68	Glu	58.32001	35.36052	175.8347	58.35703		
29	Lys	57.04432		173.49079	51.90159	30.17588		69	Leu	56.07773	38.6959	176.26812	58.00178		175.05969
30	Ser	55.16673	60.43883	172.46052	56.90605	30.35083	173.56695	70	Thr	59.76329	70.15705				
31	Thr	59.25907	68.95911	171.49865	60.31318	55.20674	172.50053	71	Gly	42.98112			59.69374	70.06683	173.5705
32	Lys	51.55462	33.67499	171.91574	59.29428	68.83618	171.53505	72	Glu			175.73564			175.87137
33	Ala	49.9478	15.48646	172.99041	51.54232	33.78305	171.96142	73	Glu	52.52676			51.58387	29.5166	172.04461
34	His	50.01483	29.84551	172.83116	49.94717	15.80754	173.02053	74	Leu	50.91474	40.24	175.04386	52.72769	26.50307	172.87986
35	Ser	55.13367	61.92613	168.80838	55.8026	29.95979	172.84747	75	Ser	55.35876	60.95851	169.96231	50.95704	40.34198	175.05143
36	Leu	52.16069	41.97934	172.01189	55.11275	61.87414	168.86141	76	Ile	55.45084			55.41201	61.00361	173.47313
37	Gln	51.68603	28.02254	173.23584	52.03115	42.16608	172.04407	77	Lys	51.02201	33.31675	171.16907	55.42283	34.61343	172.46985
38	Gly		26.32124					78	Phe	53.79133	39.95267	173.58999	51.03225	33.44625	171.22163
39	Asp	50.79425			44.98269		171.16071	79	Val	56.16243	32.59057	171.15137	53.84359	39.99493	173.64356
40	Thr	59.60358	68.31842	170.64384	50.93996	38.28121	172.25144	80	Ile	55.7845	35.44075	171.17989	56.2127	32.82044	171.1842
41	Leu	59.64512	41.44592	170.75503	68.24153	59.60886	170.68113								

^{13}C chemical shifts for the DnaA domain I amide backbone assignment. The data shown was accumulated from the following 3D experiments: CβCαNH, CβCα(CO)NH, HNCO, HN(Cα)CO, (HCα)CONH, HNCα and a Cβ optimized CβCαNH.

Suppl. Table S2

A. The chemical shift data for ^{1}H, ^{15}N.

B. The chemical shift data for ^{13}C.

Cluster 1		Cluster 2	
**DnaA Domain I**	**DnaD DDBH2 Domain**	**DnaA Domain I**	**DnaD DDBH2 Domain**
**S20**	**Y130**	**S20**	**L129**
K21	T131	P22	T131
P22	**E134**	**S23**	**I132**
**S23**	**E135**	E25	**E134**
E25	E136	**T26**	**E135**
**T26**	A138	W27	E136
W27	Q161	S30	A138
N47	**K164**	P46	D159
**E48**	H165	N47	Q161
**F49**	K168	**F49**	**K164**
R51	V170	A50	H165
**D52**	**L171**	R51	K168
**W53**	V196	**D52**	E197
**S56**	Q204	**W53**	K200
R57	Q217	**E55**	Q204
Y58	R222	**S56**	Q217
	W229	R57	W229
	L230	Y58	L230
	E231	L59	E231
	Q232	**H60**	Q232
		L61	

Suppl. Table S3

Residues of contact within the top two clusters from the HADDOCK predicted models of the DnaA domain I – DnaD DDBH2 domain interface. The residue interface was identified using a PyMOL script (https://pymolwiki.org/index.php/InterfaceResidues and https://www.schrodinger.com/suites/pymol) that applies a 1 Å^2 cutoff above the difference between the complex-based surface area and chain-only surface area to select for interface residues. AIR input residues (**Suppl. Table S1**) are displayed in bold.

REFERENCES

1. Duderstadt KE, Mott, ML, Crisona NJ, Chuang K, Yang H, Berger JM, (2010) Origin remodelling and opening in bacteria rely on distinct assembly states of the DnaA initiator. *J Biol Chem*, 285(36): 28229-28239.

2. Vranken WF, Boucher W, Stevens TJ, Fogh RH, Pajon A, Llinas M, Ulrich EL, Markley JL, Ionides, J, Laue ED (2005) The CCPN data model for NMR spectroscopy: development of a software pipeline. *Proteins*, 59(4): 687-696.
